# Computations of the shear stresses distribution experienced by passive particles as they circulate in turbulent flow: A case study for vWF protein molecules

**DOI:** 10.1371/journal.pone.0273312

**Published:** 2022-08-29

**Authors:** Oanh L. Pham, Samuel E. Feher, Quoc T. Nguyen, Dimitrios V. Papavassiliou

**Affiliations:** School of Chemical, Biological and Materials Engineering, The University of Oklahoma, Norman, Oklahoma, United States of America; Texas A&M University System, UNITED STATES

## Abstract

The stress distribution along the trajectories of passive particles released in turbulent flow were computed with the use of Lagrangian methods and direct numerical simulations. The flow fields selected were transitional Poiseuille-Couette flow situations found in ventricular assist devices and turbulent flows at conditions found in blood pumps. The passive particle properties were selected to represent molecules of the von Willebrand factor (vWF) protein. Damage to the vWF molecule can cause disease, most often related to hemostasis. The hydrodynamic shear stresses along the trajectories of the particles were calculated and the changes in the distribution of stresses were determined for proteins released in different locations in the flow field and as a function of exposure time. The stress distributions indicated that even when the average applied stress was within a safe operating regime, the proteins spent part of their trajectories in flow areas of damaging stress. Further examination showed that the history of the distribution of stresses applied on the vWF molecules, rather than the average, should be used to evaluate hydrodynamically-induced damage.

## Introduction

The distribution of hydrodynamically induced stresses on small particles, such as colloids, micelles and molecules (surfactants or proteins) that circulate in turbulent flow fields, can have an effect on their functionality. In this study, high Schmidt number particles that move due to flow advection and Brownian motion are considered, using the von Willebrand factor (vWF), a protein that circulates in the body with blood, as a case study. Changes in the configuration of the vWF concatemers can cause disease, most often related to hemostasis. In blood that flows in physiological conditions such changes do not occur, but when blood circulates through medical devices or through implanted devises like artificial heart valves, or ventricular assist devices (VADs), the conformation of the vWF molecule can change because of hemodynamic stresses. It is important, therefore, to obtain information and quantitative data, not only for the average stresses but also for the whole statistical distribution of stresses on such particles. The reasons leading to the use of medical devices is cardiovascular disease (CVD), the leading cause of mortality [1]. The projected cost of CVD in the United States for 2030, both direct and indirect, is estimated to be $915 billion, up from $314.5 billion in 2010 [2]. For patients with the most advanced stage of heart failure (HF), it has often been necessary to resort to medical devices, like VADs, to improve blood circulation [2–4], while patients with heart valve disease often need to receive artificial valve implants [5]. However, both VADs and artificial valves are associated with device-related hemostatic complications that commonly affect patients’ recovery and life quality after implantation.

The vWF is a long polymeric protein with 2050-residue monomers linked head-to-head and tail to tail into concatemers of up to 200 monomers [6–8]. If the protein molecule undergoes damage because of shear flow (e.g., cleavage, or changes in the way the protein folds) then its efficacy is diminished, leading to a blood disorder [9–13], called the Von Willebrand disease (vWD). Frequent nosebleeds, easy bruising and difficulty to form thrombus after a small scrape or cut are common symptoms for people with vWD. This common disorder affects up to 1% of the US population, and 20–30% of patients with VADs [13]. There is a critical shear stress beyond which vWF starts to deform at about 5 Pa [9].

Computational modeling of the vWF has focused mainly on coarse-graining approaches, since molecular dynamics are not practical for modeling molecules of size as large as the vWF. In coarse graining, the atoms that form the vWF molecule are grouped in clusters or beads, and the interaction between such clusters is calculated as the simulation advances in discrete time intervals [14, 15]. Such modeling is usually focused on the molecular interactions between the vWF and the surrounding fluid or the adhesion process of the vWF to the vascular vessel walls. The microenvironment around the vWF can be thus modeled, but the behavior of a large number of molecules in a blood flow domain is not practical with coarse graining.

Recently, there have been several studies focusing on the unraveling and cleavage of vWF in shear and elongational flow. Fu et al. pointed out that the activation of vWF underwent two-step conformational transition: elongation from compact to linear form, and a tension-dependent local transition to a state with high affinity for GPIbα [12]. Carlo et al. carried out experiments to separate the effects of shear stress from the effects of ADAMTS-13 and found that ADAMTS-13 cleavage is distinct from shear stress for vWF degradation. When the purified vWF protein was exposed to supraphysiologic shear stress, high-molecular weight vWF multimers degraded into low molecular weight multimers but the amount of generated vWF was not in large quantities [16]. Bortot et al. have shown that the cleavage of vWF as facilitated by the turbulent flow conditions leads to the functional deficiency of vWF [17]. Sharifi et al. adopted the scission theory for polymers on biological multimers providing an understanding of flows producing strong extensional forces on vWF and resulting to cleavage, especially in turbulent flow [18].

In the present work, direct numerical simulation (DNS) was used to obtain the details of the flow field in biologically relevant turbulent and transitional flows. A Lagrangian method for tracking the trajectories of vWF surrogate particles in space and time was applied. It should be very clear that the molecular structure of the vWF was not obtained with this numerical approach, but the contribution of our work was to show with detailed calculations the level of hydrodynamic stresses and their distribution on particles that move the same way as vWF molecules would move in a turbulent flow field. It is not feasible to conduct molecular dynamics in Reynolds number flows comparable to the ones applied herein, which are representative of the Reynolds number in cardiovascular devices, as it is not feasible to conduct molecular computations for the time duration presented herein. We also offer result for plane Poiseuille flow and for Poiseuille-Couette flow. As Bortot et al. have pointed out, most prior studies (both experiments and computations) have been conducted under laminar flow conditions, even though turbulence is needed to cause severe damage to vWF [17]. In the present work progress toward covering this knowledge gap is reported–the shear stress along the particle trajectories was calculated providing not only average stresses, but the distribution of stresses. Further, the determination of stresses when particles were injected at different locations in the flow field was possible. While the computations were conducted using dimensionless equations, the results were related to cases of actual biomedical devices. Furthermore, the effects of the history of particle motion were explored. In prior work, we have examined the history of extensional stresses [19], but the focus herein was exclusively on shear stresses and on probing the idea that in turbulent flow fields predictions of average stresses or wall shear stresses are not enough to predict the levels of protein damage due to shear stress.

### Computational approach

We employed an in-house DNS to solve the Navier-Stokes equations in a channel without using any turbulence modeling. This DNS has been verified with experimental results and has been employed in prior publications [19–21]. The current studies not only cover Poiseuille flow (PF) but also Poiseuille-Couette flow (PC) in the transitional regime. We choose the PC flow for simulation because it occurs in left ventricular assist devices (LVAD), where rotating parts contribute a Couette character of the flow and the pressure drop contributes a Poiseuille flow character to the flow. We wanted to examine different types of flows, and Couette flow serves this purpose with its different structure close to the wall than Poiseuille flow. The coherent turbulent flow structures are larger in Couette flow, and the time and length scales of these flow structures differ from PF even at the same Reynolds number [22]. In addition, the Reynolds number for the PC flow field in our study is in the transition range, not in the fully turbulent flow regime. Therefore, results for two cases, one corresponding to blood pumps with fully turbulent flow and one corresponding to LVADs, can be provided.

The simulations were conducted in dimensionless units, using the kinematic viscosity of the fluid, *ν*, and the friction velocity of the fluid, u^*^, to normalize the Navier-Stokes equations. The numerical scheme has been described in details elsewhere–it is based on a pseudospectral method that employs Fourier transforms for the velocity in the periodic streamwise and spanwise directions, and Chebyshev polynomial expansions in the wall normal direction. The rotational form of the Navier-Stokes equation is solved, with the Marcus correction for the calculation of the pressure [23, 24]. The dimensions of the computational box in the *x* (streamwise), *y* (wall-normal) and *z* (spanwise) directions were *30πh* x *2h* x *4πh* for the PC flow and *16πh* x 2*h* x2*πh* for the fully turbulent PF, where *h* was the half-channel height. The flows were periodic in *x* and *z*, with no-slip boundary conditions at the channel walls. The bottom wall of the PC flow channel was moving in the negative x direction with velocity 7.78 in wall units, and the top wall was moving with the same velocity but in the positive x direction. The number of mesh points was 512x128x128 for the PC flow and 1024x256x128 for the PF. Since the goal here is not to explore the rheological behavior of blood, but the values of the stresses in a flow field, the assumption of a Newtonian fluid at high shear rates was reasonably made [25–27]. The time step for the simulations was Δ*t* = 0.075 for the PC flow and Δ*t* = 0.1 for PF.

The friction Reynolds number, *Re*_*τ*,_ was 80 for the PC flow and *Re*_*τ*_ = 300 for the fully turbulent PF. VADs operate in rather low Reynolds numbers [28, 29] with PC flow in the annular space between the rotor and the shell of the VAD device. Typical annular spacing in axial flow VADs is in the range from 1mm to 5mm [30–33]. Choosing a PC with channel width equal to 1.5 mm for *Re*_τ_ = 80 and assuming blood viscosity μ = 0.0035 Pa s, the shear stress at the wall, *τ*_w_, was calculated to be 35.16 Pa, with friction velocity u* = 0.183 m/s. The Reynolds number for turbulent PF can be related to the Reynolds number for flow in the typical centrifugal blood pump that was offered as part of the Food and Drug Administration critical path initiative for simulations [25, 34–36]. The case of flow of blood at 7 lt/min at Re = 3661 in a 1.5mm diameter pipe corresponds to a mean velocity of 1.990m/s, which when compared to the mean velocity of 16.68 in viscous wall units of the *Re*_*τ*_ = 300 simulation gives u* = 0.1208 m/s and *τ*_w_ = 14.95Pa. Note that the friction velocity and the shear stress at the wall are related as u* = (*τ*_w_/ρ)^1/2^, where *ρ* is the density of the fluid (*ρ* = 1050kg/m^3^ for blood). Table 1 is a summary of the simulation conditions and the scaling factors for transforming the values presented herein from viscous wall units to dimensional quantities for the PC and the PF cases described above. Based on Table 1 the computational domain for the PF is 37.70 x 1.5 x 4.71 mm^3^ and for the PC flow it is 70.78 x 1.5 x 9.45 mm^3^.

**Table 1:** Parameters of the simulations for Poiseuille and Poiseuille-Couette flow and scaling information for transforming quantities in viscous wall units to dimensional quantities.

Parameters	Poiseuille flow (PF)	Poiseuille-Couette flow (PC)
Computational box size	*16πh* x *2h* x *2πh*	*30πh* x *2h* x *4πh*
Computational box mesh	1024 x 256 x 128	512 x 128 x 128
Time step, Δ*t*	0.1	0.075
Viscous time scale, t*	2.1x10^−5^ s	5.13x10^−5^ s
Viscous length scale. l*	2.5x10^−6^ m	9.4x10^−6^ m
Friction Reynolds number, *Re*_*τ*_	300	80
Computational box size	*16πh* x *2h* x *2πh*	*30πh* x *2h* x *4πh*
Friction velocity, u*	0.1193 m/s	0.183 m/s
Wall shear stress, *τ*_*w*_	14.95 Pa	35.16 Pa
Position of release, Y_0_	1.5, 3, 5, 10, 15, 75, 300	0, 3, 5, 15 and 80
Channel width	1.5 x 10^−3^ m	1.5 x 10^−3^ m
Number of particles	800,000	500,000

Lagrangian scalar tracking (LST) of mass markers that represented vWF molecules dispersing in the flow field was conducted after the flow reached stationary state using the particle tracking algorithm of Kontomaris et al. [37]. These markers were assumed to be passive, which we define as particles whose trajectories are affected by the flow field and by their Brownian motion, but their presence in the flow does not affect the velocity of the surrounding fluid. While the particles may change internal conformation under stress (such as vWF molecules would), they were assumed to continue to not affect the flow field around them. Prior computational work has also made the assumption that vWF is a passive particle [17].

Mass markers were injected instantaneously at different locations in the flow, at *Yo* = 0, 3, 5, 15, 80 wall units away from the bottom channel wall for the PC flow and at *Yo* = 0, 1.5, 3, 5, 10, 15, 75, 300 for the PF simulation. Possible bias because of the initial velocity field was mitigated by releasing markers from 20 lines spanning the *x-z* plane at every *Yo* value. These lines were placed along the *z* direction and were uniformly spaced along the *x* direction, with the first line starting at *x* = 0. We injected 100,000 markers at every *Yo* from locations that were equally spaced in the spanwise direction, *z*. The streamwise location reported herein was calculated in relation to the point of origin of each marker and was calculated by subtracting the initial position *x*_0_, as (*x*–*x*_0_). The position vector designated as $\vec{X}(\vec{x_{0}},t)$ corresponded to the position of each marker, given that this marker was released at position $\vec{x_{0}}$ at time t = t_0_ = 0. The velocity of this same marker in the Lagrangian was $\vec{V}(\vec{x_{0}},t)$, and it was related to the Eulerian velocity, $\vec{U}$, of the fluid at the marker location as $\vec{V}(\vec{x_{0}},t)=\vec{U}[\vec{X}(\vec{x_{0}},t),t]$. The markers moved because of convection in the flow field, using the relation

$$\vec{V}(\vec{x_{0}},t)=\frac{\partial \vec{X}(\vec{x_{0}},t)}{\partial t}$$ (1)

and because of molecular diffusion. The convection was simulated by integrating Eq (1) with a second order scheme (Adams-Bashforth) and the diffusion was simulated by adding a random jump of each marker at the end of the convective motion [19, 38]. The random movement was determined by random selection of the particle jump utilizing a Gaussian statistical distribution. According to the Brownian motion theory developed by Einstein, the random jump should follow a Gaussian probability density function that has an average value of zero and a variance that depends on the diffusion coefficient of the particle. Utilizing the Schmidt number, *Sc = v/D*, where *D* is the molecular diffusivity of the vWF in blood, the standard deviation, σ, for the Brownian motion in each space direction was found as $\left(\sigma =\sqrt{2\Delta t/Sc}\right)$, where Δ*t* is the time step of the simulation. Assuming that the hydrodynamic radius of a vWF molecule was 1.8 x10^-8^ m (based on a molecule with molecular weight of 20,000KDa [39]), and that the dynamic viscosity of blood was 0.0035 Pa s, the Stokes-Einstein relationship produced a diffusivity coefficient *D* = 3.6119x10^-12^ m^2^/s, and a Schmidt number of 914,179 (the blood kinematic viscosity was taken as *ν* = 3.30189x10^-6^ m^2^/s). In order to be able to use the Stokes-Einstein equation, the vWF particles were assumed to be spherical. Possible changes of the vWF shape in the flow field were not accounted for the diffusivity calculations. The use of a particle diameter in the Stokes-Einstein equation was justified by the assumption that the most important contributions to the motion of the vWF were convection and Brownian motion, while drag effects were not important. Prior researchers have also assumed that the vWF can be treated as a sphere [18, 40]. Others have implied in their analysis that the vWF is spherical, for example Pushin et al. [41] assumed that the forces acting on the vWF could be calculated as stress multiplied by the area of a circle (i.e., the circle is the projected area of a sphere on the stress plain). Further, it is known that when mechanical stresses are low, interdomain attraction between the vWF molecular regions lead to a collapsed conformation [42]. Therefore, instead of updating the protein diffusivity of the trajectories of modified proteins as they might unfold in flow, it was assumed that small changes in the protein size (within an order of magnitude), would have negligible effects on the probability density functions for stresses. The justification for this was based on prior results regarding the dispersion of high *Sc* particles in turbulent flow. We have seen that particles with *Sc* > 2,400 behave quite the same in terms of statistical quantities, such as the mean particle velocity, the Lagrangian autocorrelation coefficient, and the rate of dispersion [20, 43].

Since the velocity of the vWF markers was assumed to be equal to the velocity of the fluid at the marker location, it was assumed that the shear stress acting on each marker was equal to the shear stress based on the Eulerian velocity at the marker’s position in the Eulerian framework. In dimensionless form, the shear stress was calculated as $\tau^{+}=\frac{du_{x}^{+}}{dy^{+}}$, where $u_{x}^{+}$ is the streamwise velocity in wall units and *y*^+^ is the dimension normal to the channel wall. The transformation to dimensional stress was done by multiplication of the dimensionless stress by the scale *τ*_w_, appearing in Table 1. This was the largest component of the stress tensor acting on the particles, and this is what is reported from now on in this manuscript. Statistical analysis to find the average stress as a function of time and to calculate the probability density function followed, once the statistical sample space from the stress on each of the 100,000 individual particles was calculated at each time step and for each location of particle release.

The history of shear stresses was calculated using a Lagrangian integration of the shear stress components. The time integral of the shear stress along the trajectory of each particle was calculated, and then the distribution of the Lagrangian integral was determined as a function of time. The effects of the history of the stresses on the hemolysis of red blood cells have been calculated with the same approach [25, 44, 45]. Investigating the history of the stress on each molecule is important, since there is evidence that the vWF can stretch or unfold under stress, even when it is exposed to stress for small time intervals [41]. These deformations have been reported to be irreversible. Small stresses applied for long time periods can result in cleaved or deformed vWF, as can high stresses experienced for shorter time periods.

## Results and discussion

### Transitional Poiseuille-Couette flow

**Fig 1:**
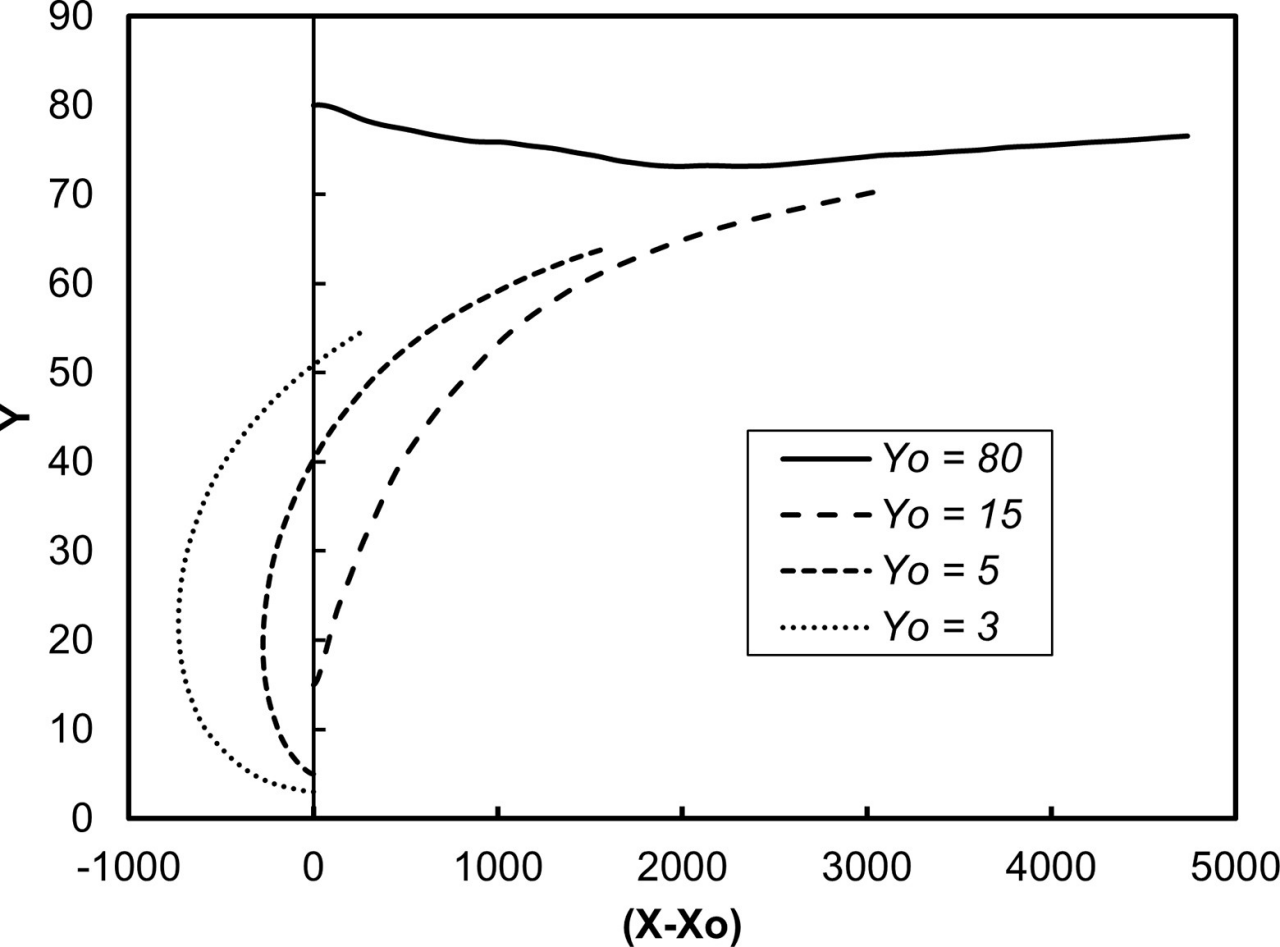
Average position of the vWF markers in the Poiseuille-Couette velocity field. Results for particles released within the viscous wall layer (*Yo* = 3 and 5), within the buffer region (*Yo* = 15) and at the center of the channel (*Yo =* 80) are presented.

**Fig 2:**
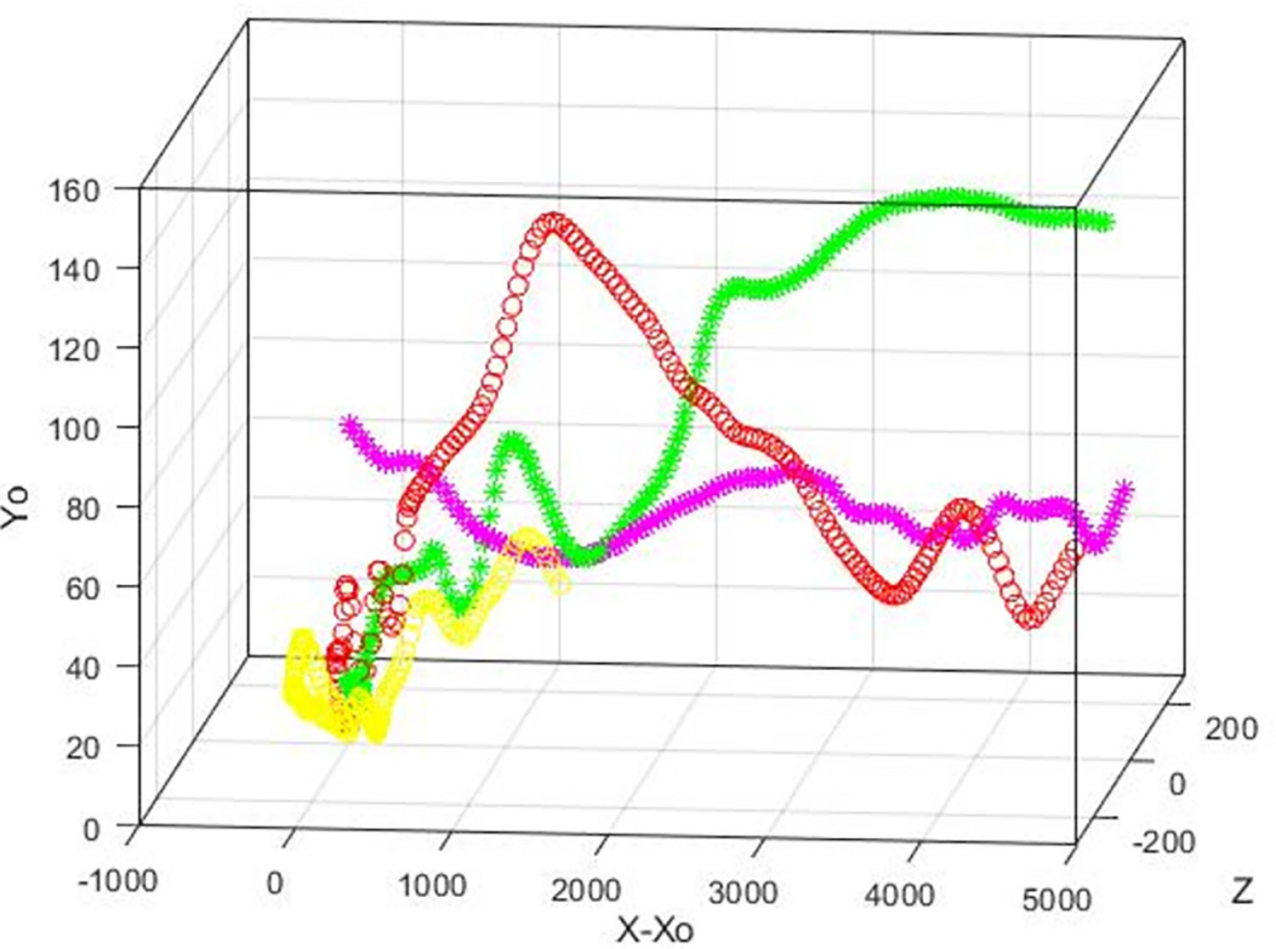
Trajectories of individual vWF markers in the Poiseuille-Couette velocity field. Examples of particles released at *Yo* = 3 (yellow circle), *Yo* 5(red circle), *Yo* = 15 (green start), *Yo* = 80 (pink star) are shown.

Fig 1 is a display of the average location of the vWF surrogate particles as they moved in the flow field. This is the trajectory of the center of mass of the cloud of the particles as a function of time in the *X*, *Y* space, where *X* is the average (*x*-*x*_o_) position of all particles released at the same distance *Yo* from the channel wall, and *Y* is the average *y* position of these particles. The averages were calculated at each time step of the simulation, from time 0 to 300 in wall units. The bottom wall of the channel was moving in the negative *x* direction and the top wall in the positive *x* direction, so the average particle position was in the negative *X* region at early times. The Poiseuille part of the motion started to be effective in moving the particle cloud as the particles dispersed in the flow field at later times. For particles released at the center of the channel, the Poiseuille effect dominated and the X values were positive. One would need to keep the data in this figure in mind when discussing the stresses, because the stress distribution was affected by the location of the particle cloud. Fig 2 is an illustration of the trajectories of individual particles released at different locations in the computational box.

**Fig 3:**
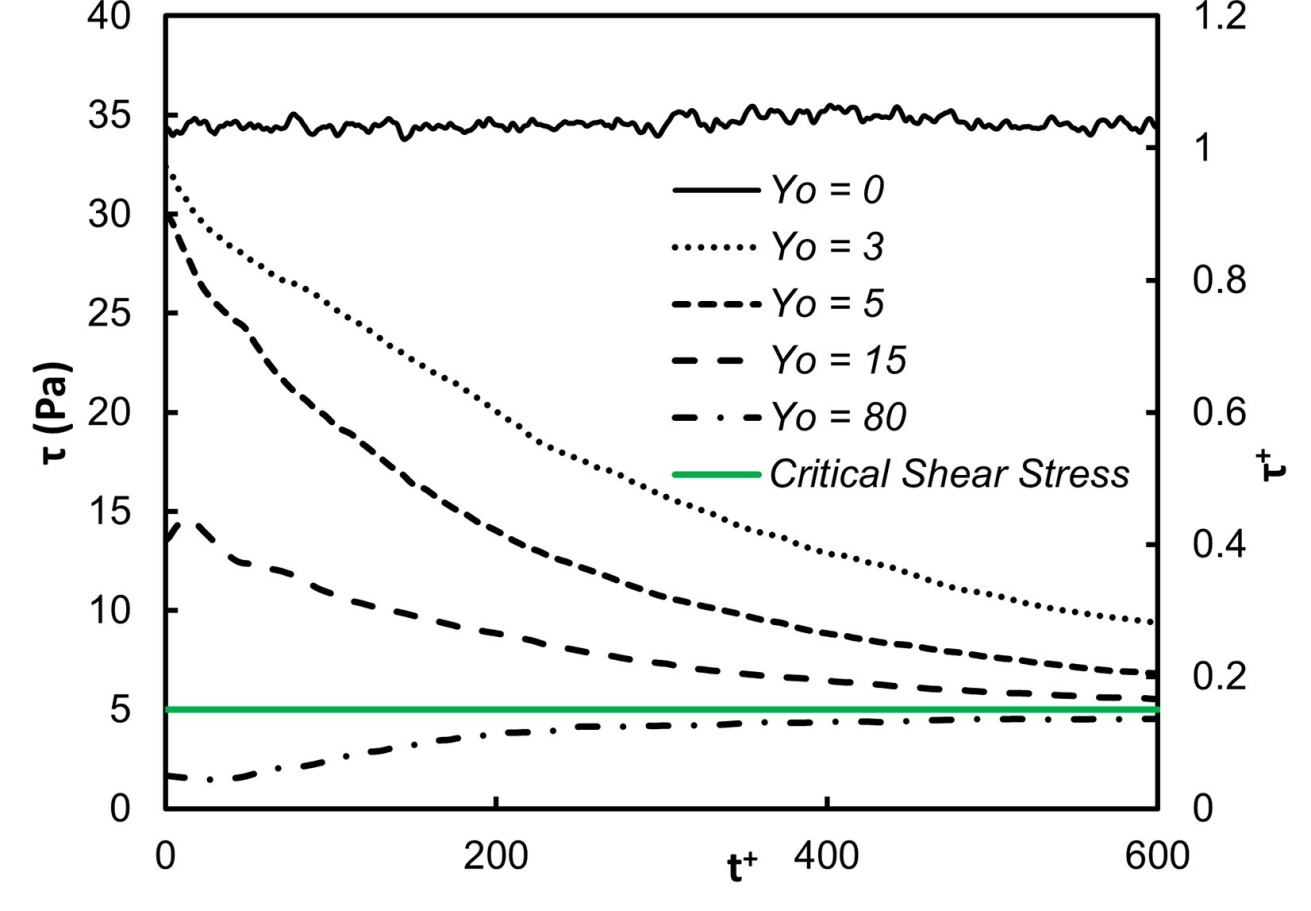
Average of absolute shear stress as a function of time for vWF particles injected at different initial locations. The abscissa in dimensionless viscous parameters and in dimensional units is shown.

**Fig 4:**
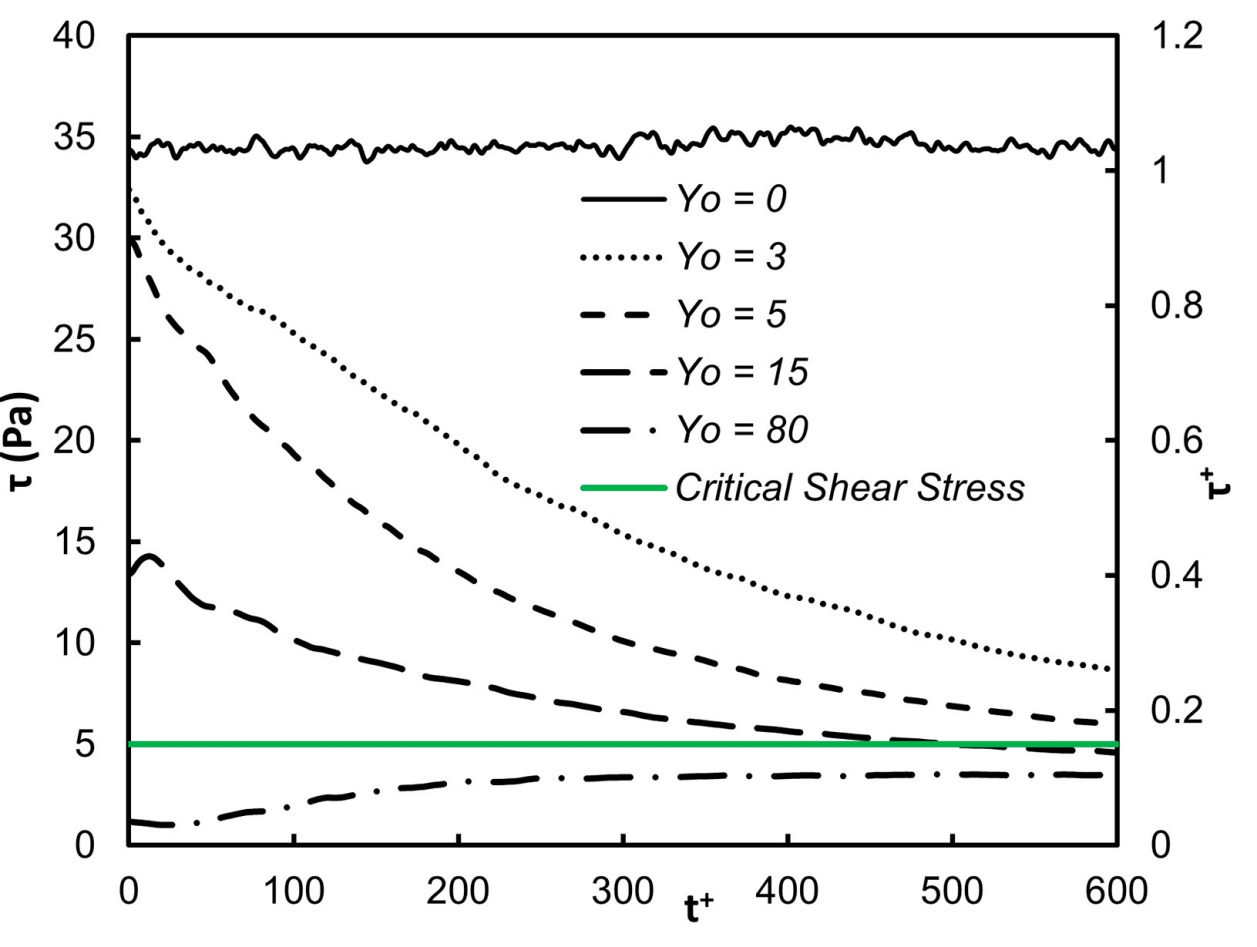
Average of actual shear stresses. The abscissa in dimensionless viscous parameters and in dimensional units is shown. One can adjust the dimensional stress axis at the same *Re* by considering a new channel width with a new corresponding mean velocity to recalculate *τ*_w_ and u*, and using the dimensionless values presented.

The average shear stress on the vWF particle locations as a function of time is presented in Figs 3 and 4. The stress was the highest close to the wall, as expected. The shear was dominated by the mean velocity gradient, which in viscous wall units has the value of one by definition. Since the vWF particles mostly stayed in this region, the average stress did not change dramatically over 600 time units. However, when the vWF injection was farther away from the wall, the average stress showed variation. As the average position of the particles moved away from the wall (see Fig 1), the average stress took lower values. For release at the buffer region, *Yo* = 15, the average stress was roughly 40% of the maximum value, and for release in the center of the channel the stress was much lower–about 10% of the maximum value. However, when one considers the dimensional value of the stress, it was higher than 5 Pa–the critical stress that can cause damage to the vWF molecular configuration. Fig 3 is a plot of the average of the absolute value of the stress. In this way, whether the vWF underwent stress in the positive or negative *x*-direction is not important. Average of the stress by accounting for positive and negative stresses is shown in Fig 4.

**Fig 5:**
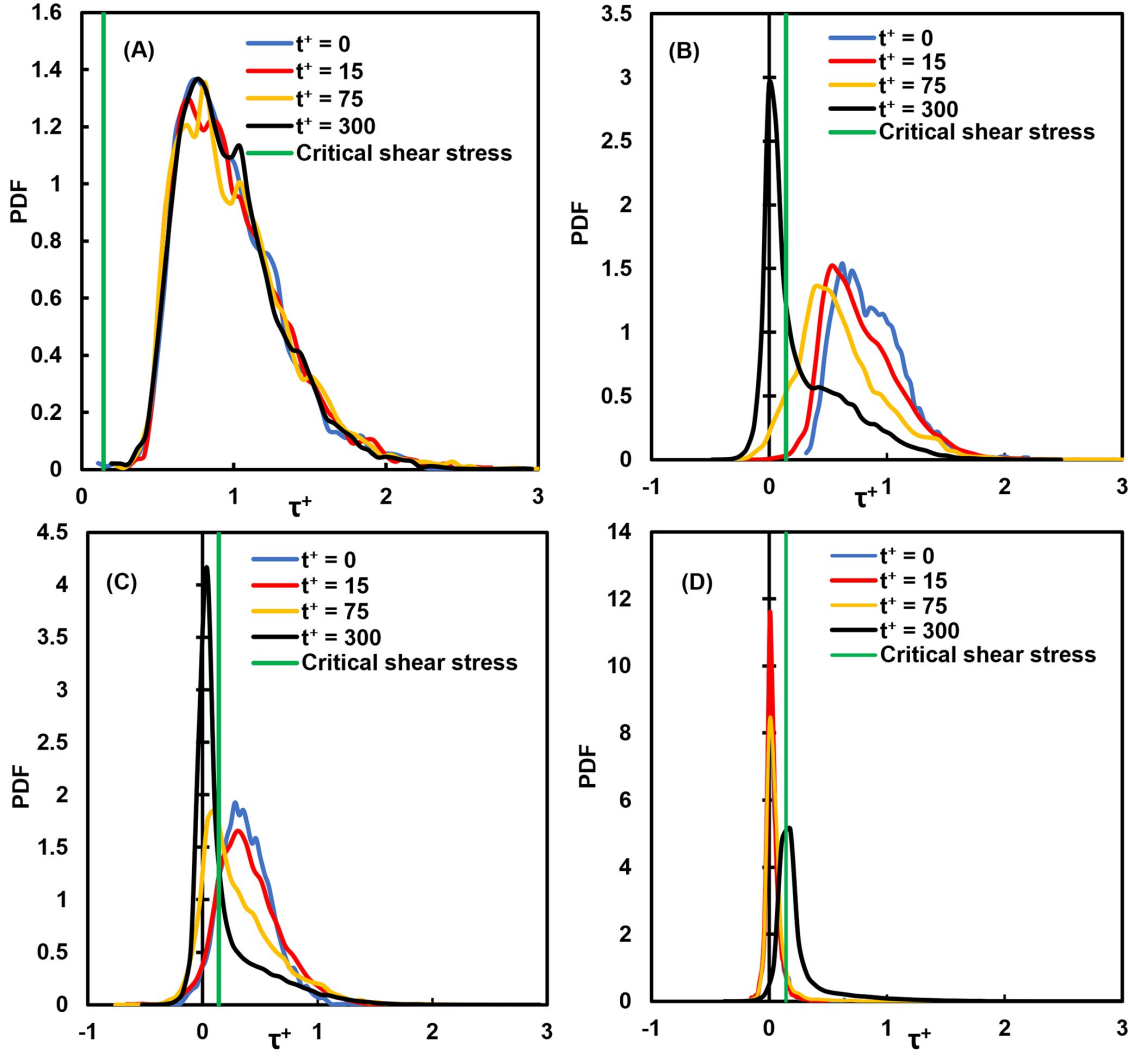
Distributions of shear stress on vWF particles at different times in viscous wall units. **(A)** Cloud released at the channel bottom wall, **(B)** release at the edge of the viscous wall subregion (*Yo* = 5), **(C)** release within the buffer region (*Y*o = 15) and **(D)** release at the channel center.

One of the questions that this paper aims to answer is whether the average stress provides a complete picture of the stresses that the vWF molecules undergo in this flow field. The full distribution of stresses is presented in Fig 5 at specific time instances after particle injection. While the stress distribution for release at the channel bottom wall did not change much with time, as seen in Fig 5A, when the release point for the vWF was at a distance from the wall, the distribution changed. As the particles moved on average away from the wall after their release (see Fig 1), the probability density function (PDF) for the stress distribution increased its variance (it became wider). The particles did not all undergo the same stresses, but they spent some time in areas of high stress (higher than the average) and some time in areas of low stress. The stress on particles released at the center of the channel followed a distribution that was narrower for all times. This is expected, when one considers the average particle position as seen in Fig 1. The mean velocity gradient was not as large as in other regions of the channel, resulting in a more symmetric distribution (Fig 5D).

The injection location was important in determining what stress a vWF particle would undergo as it moved in the flow field. It was also apparent that a particle would experience different stresses depending on its trajectory. Thus, the history of stresses was not the same for every particle in the flow. In other cases of blood flow, the history of stresses on particles, specifically red blood cells (RBCs) is important and can lead to cell trauma. The well-known phenomenon of RBC hemolysis has been modeled with power law models that incorporate the level of stress and the time of exposure of RBCs to such stresses. Furthermore, data by Zhussupbekov et al. [46] indicate that the unfolding of vWF also follows the power law model. In their study, a constant percentage of vWF unfolding occurred either when the stress was high and the time of exposure was low, or when the inverse occurred. Other indirect evidence by Heidari et al., [14] showed that the change in protein conformation with time and shear rate follows a power law model. In Fig 3 of their work, they plotted the radius of gyration of the vWF versus a characteristic time scale multiplied by the shear rate.

**Fig 6:**
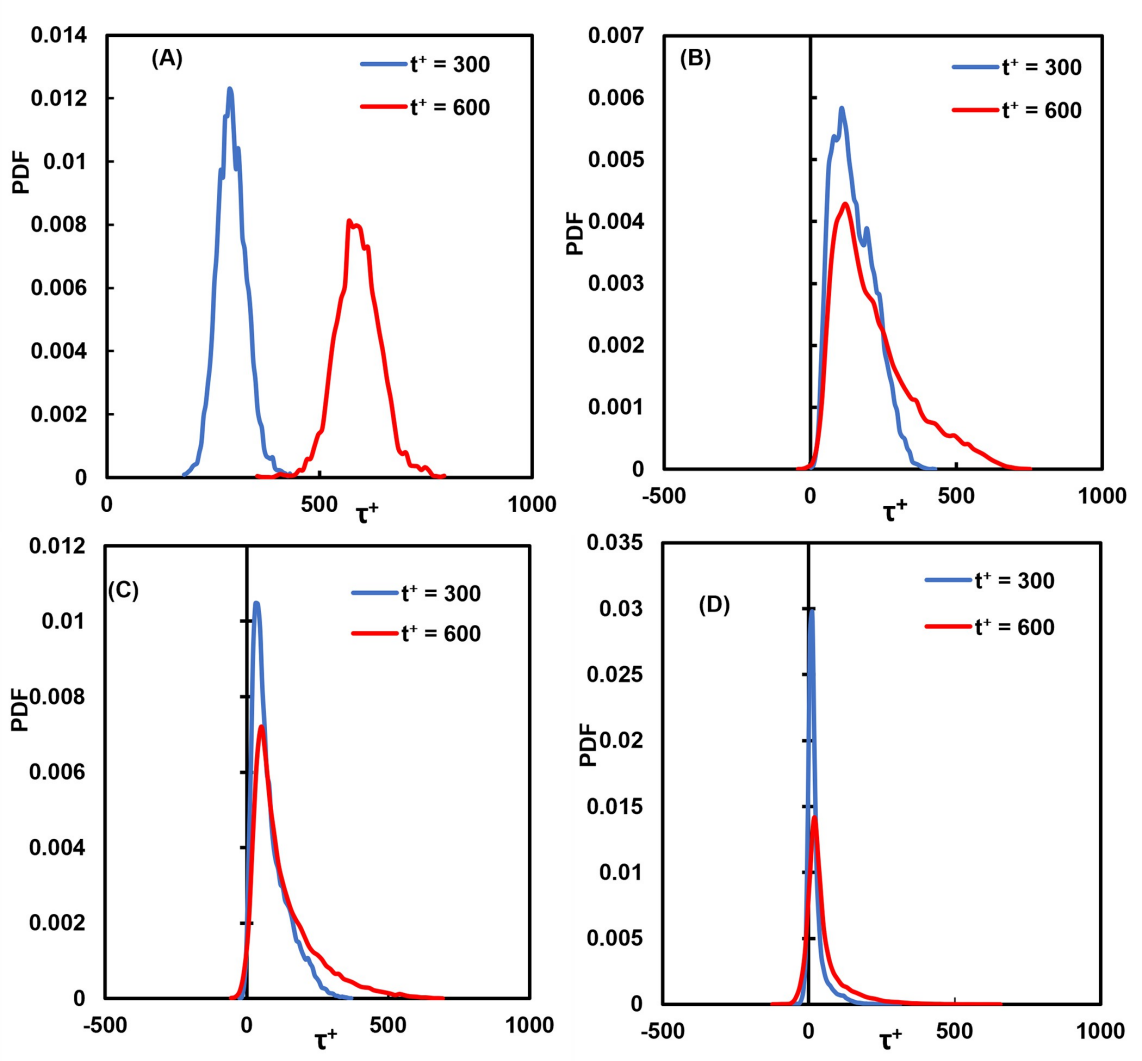
Distributions of the shear stress history for vWF particles at different times in viscous wall units. **(A)** Cloud released at the channel bottom wall, **(B)** release at the edge of the viscous wall subregion (*Yo* = 5), **(C)** release within the buffer region (*Yo* = 15) and **(D)** release at the channel center.

Our hypothesis was that the vWF undergoes a similar process–the longer it was exposed to high stresses, the higher the probability of reconfiguration and damage. It is important, therefore, to investigate the history of stresses and observe the distribution of stress histories. A power law model is a reasonable approximation for modeling the effects of time of exposure to stress, even though the details of the mechanism of vWF cleavage and elongation are not explicitly modeled. The power law exponents are not known, so the calculations herein were carried out with the assumption that the exponents are equal to one. The distribution of the history of the stress on particles at two different times after their injection is presented in Fig 6. The integrated stress became larger over time, and the distribution was displaced to larger values as time advanced. Further, the variance of the distribution increased, indicating that there were particles that underwent much higher stresses than others. For example, there were particles that underwent much higher stresses while traveling in the same flow field, even when released form the same location. In Fig 6B, as an example, some particles experienced an integrated stress of 100 τ^+^, and others saw 500 τ^+^ or more (recall that multiplying by 35.16 Pa yields the integrated stress in SI units). In all instances, the vWF particles experienced high stresses, above the critical value of 5 Pa [9].

### Fully turbulent Poiseuille flow

**Fig 7:**
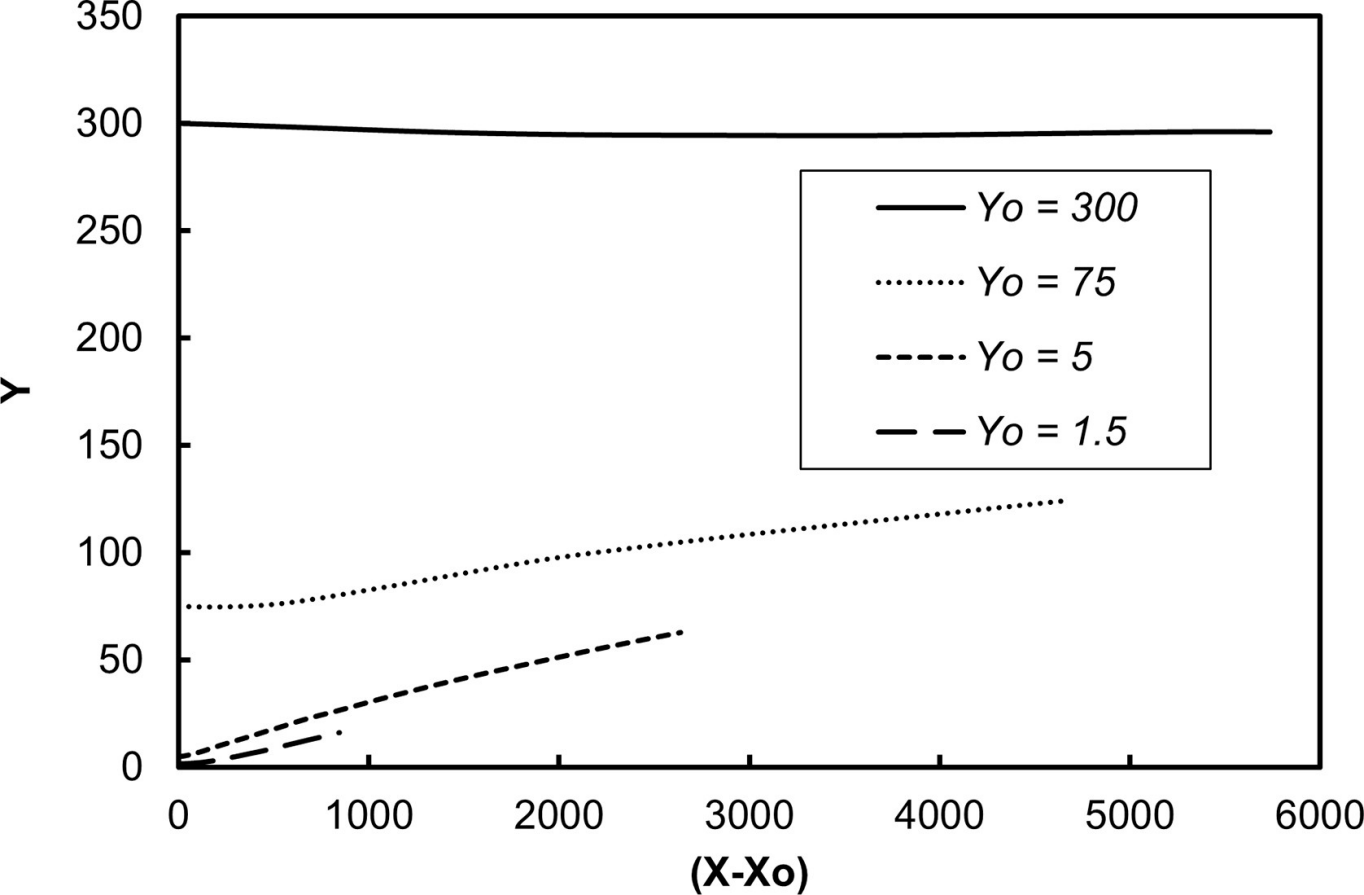
Average vWF position in fully-developed Poiseuille turbulent flow. Results for particles released within the viscous wall layer (*Y*o = 1.5 and 5), within the logarithmic layer (*Yo* = 75) and at the center of the channel (*Yo* = 300) are presented.

**Fig 8:**
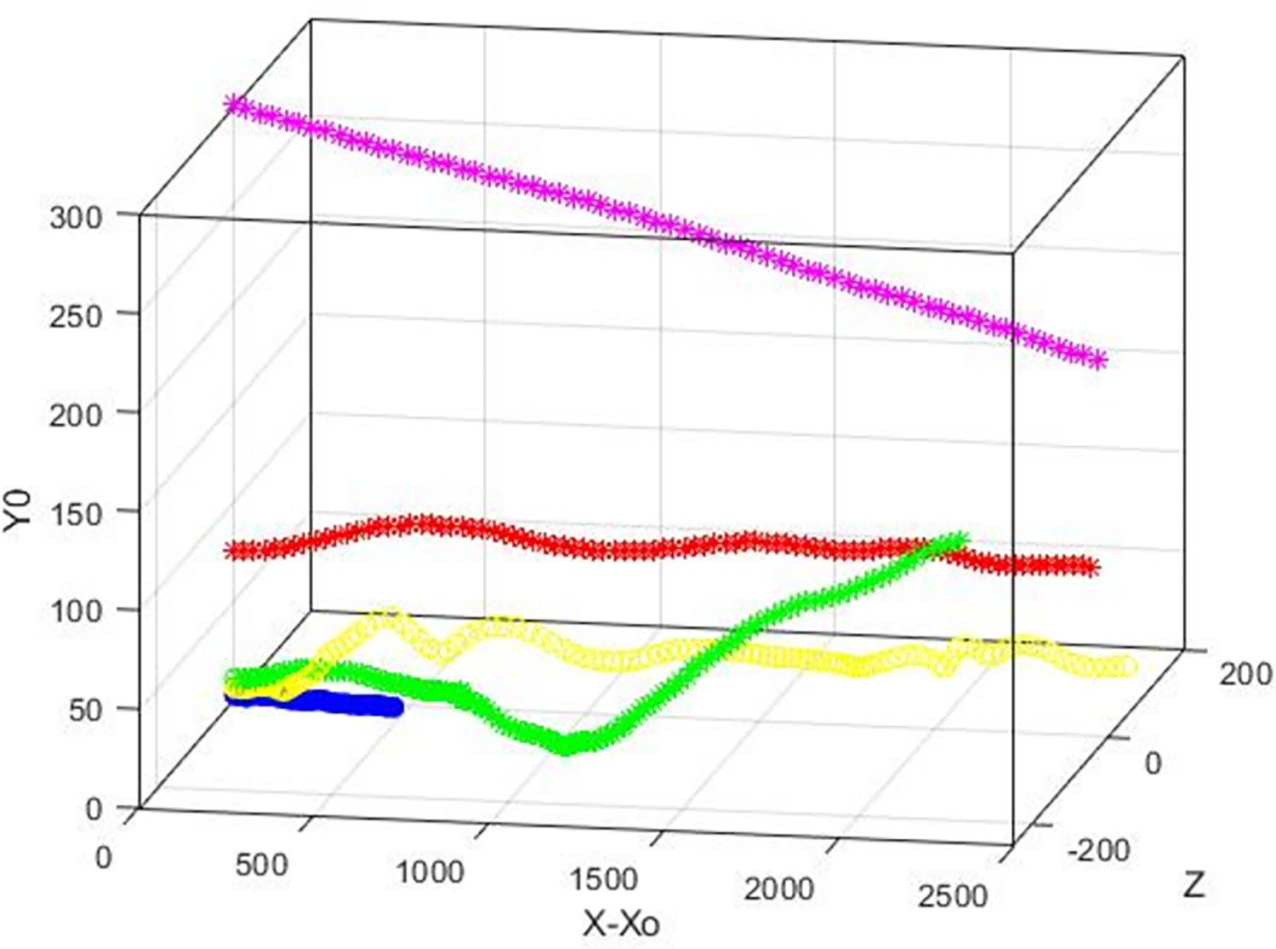
Trajectories of particles released in a Poiseuille flow field. Examples of particles released at *Yo* = 1.5 (blue circles), *Yo* = 5 (yellow circles), *Yo* = 10 (green stars), *Yo* = 75 (red stars), *Yo* = 300 (pink star dot).

In medical devices that help with blood circulation outside the body, like dialysis machines, the flow can be in the turbulent regime. Fig 7 is a display of the average location of the vWF surrogate particles as they moved in a fully turbulent flow field and Fig 8 is an illustration of trajectories of individual particles released at different locations. The average position of the vWF cloud after injection at different locations in the channel provided information for the particle trajectories on average. Since there was no Couette component of the flow, the particles moved in the positive *x* direction. Particles released at the center of channel (*Yo* = 300) did not have a reason to move above or below the center (on average), but particles released closer to the wall tended to move on average away from it, because of the boundary that the wall presented to them. Closer to the wall, the mean velocity of the fluid in the *x* direction was smaller, and the extent by which they moved in the *x* direction was mostly affected by the magnitude of the mean flow in the streamwise *x* direction.

**Fig 9:**
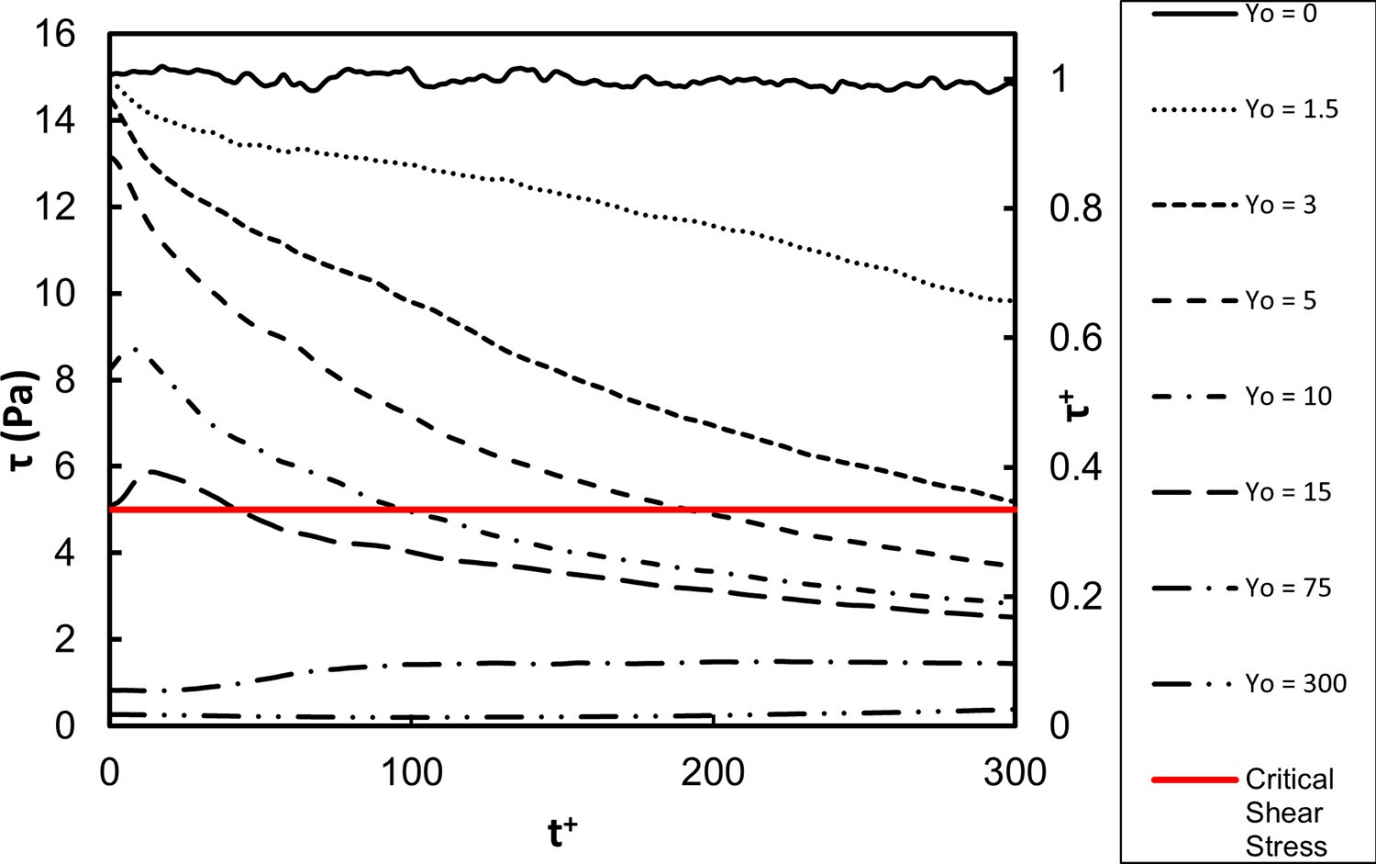
Average of the absolute value of the shear stress as a function of time for vWF particles released at different initial locations in fully turbulent flow. The abscissa in dimensionless viscous parameters and in dimensional units is shown.

**Fig 10:**
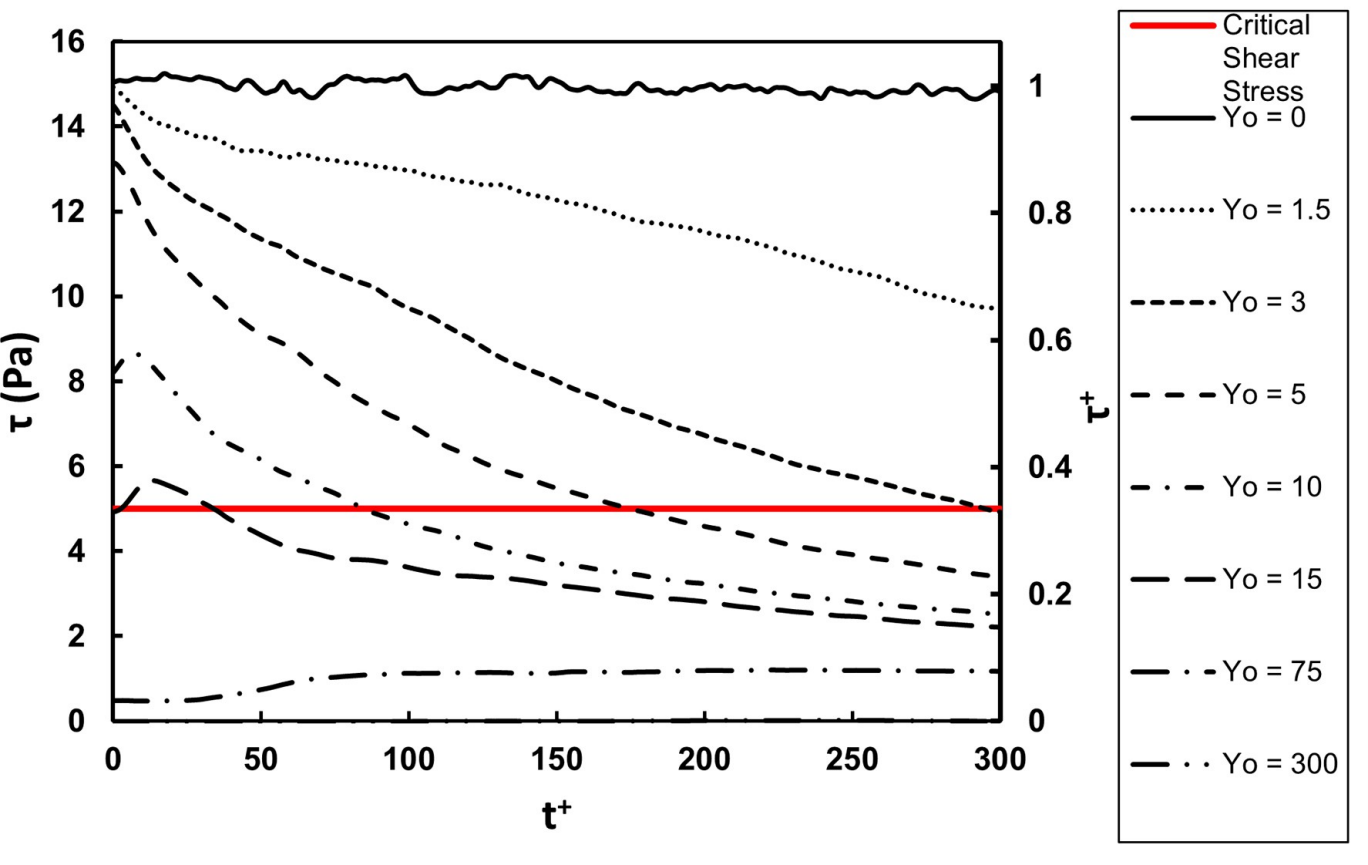
Average of actual shear stresses. The abscissa in dimensionless viscous parameters and in dimensional units is shown. The dimensional stress axis can be adjusted for a different channel when the actual mean velocity and channel size is known by using the dimensionless values presented.

**Fig 11:**
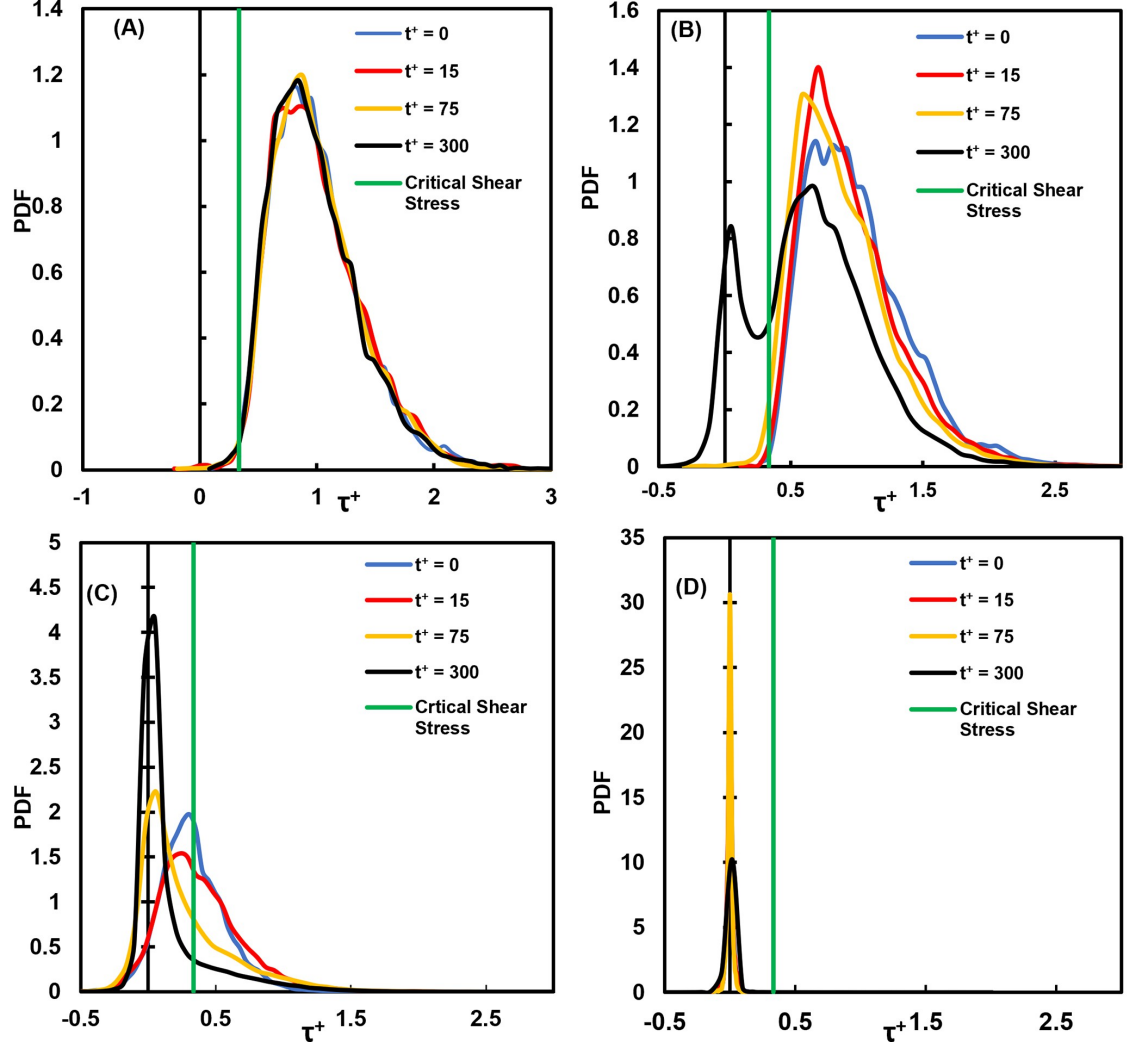
Distributions of shear stress on vWF particles at different times in viscous wall units. **(A)** Cloud released at the channel bottom wall, **(B)** release within the viscous sublayer (*Yo* = 1.5), **(C)** release within the buffer region (*Yo* = 15) and **(D)** release at the channel center.

The average of the absolute value of the shear stress on the vWF particle locations as a function of time is presented in Fig 9. The average of the actual shear stress is shown in Fig 10, while the actual stress distribution at different times is presented in Fig 11. The qualitative behavior of the average stresses as a function of the location of particle injection was similar to the case of Poiseuille-Couette flow. The thick green line indicating the critical shear stress for vWF damage crosses through the stress profiles. Depending on the location of release, and on the time elapsed since the release, the particles move from regions with stresses below the critical value to regions above the critical point and vice versa. This was even more pronounced when examining the full distributions of the stresses. There was a probability that a vWF particle would experience below or above critical stresses, depending on the time since release and depending on the location of release.

**Fig 12:**
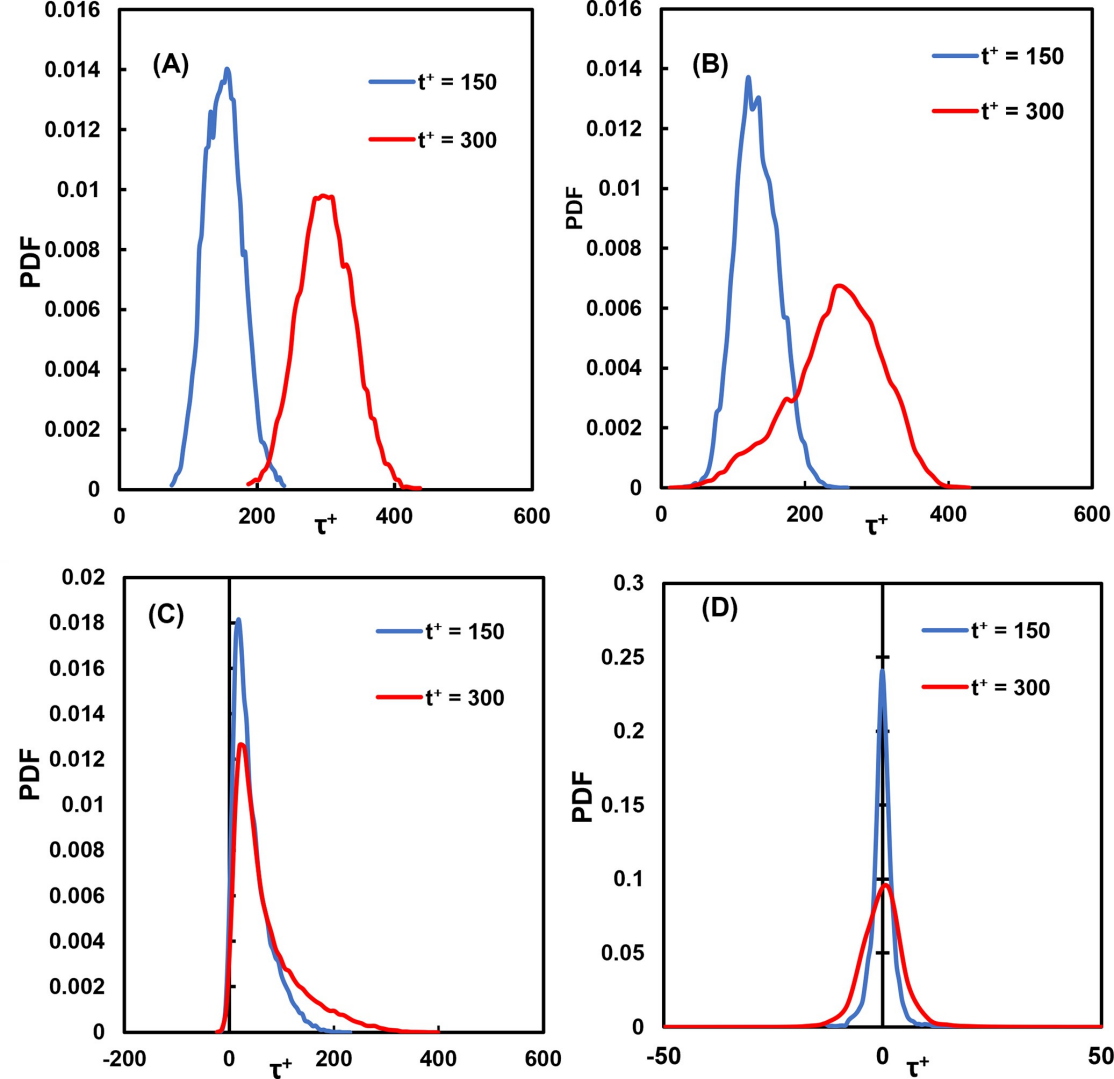
Distributions of the shear stress history for vWF particles at different times in viscous wall units. (**A**) Cloud released at the channel bottom wall, (**B**) release within the viscous wall subregion (*Yo* = 1.5), (**C**) release within the buffer region (*Yo* = 15) and (**D**) release at the channel center.

The history of stresses on a particle was also important in this case. As seen in Fig 12, as time advanced, the distribution of the history of the stresses increased in its variation. The vWF particles did not all have the same exposure to damaging stress, but instead experienced a range of stresses that became wider as time advanced.

## Conclusions

The distribution of shear stresses induced on vWF surrogate particles injected *in silico* to computationally obtained velocity fields that are found in blood flow through biomedical devices and implants was calculated in detail. A Lagrangian approach, where individual vWF particles were followed in time and space while monitoring the hydrodynamic stresses, was applied. With this approach, details on the stresses and their distribution as a function of the location of particle release and the type of flow (transitional and fully turbulent) were obtained. It should be noted here that the model for the vWF is a simplification of an actual medical device, in the sense that the interactions between the vWF molecules and the surrounding beyond hydrodynamics are not considered, for example interactions between vWF proteins that come in proximity to each other and might get entangled. However, an interaction like that, in the framework utilized herein would mean that the Schmidt number of the entangled particle would now be different, but the trajectory that the new particle would follow and the stresses seen by this new particle would not be different than what is reported here. One can improve upon the present model by incorporating a bead-and-spring model for the vWF molecule and modeling the configuration and orientation of the molecule under stress. Such an approach could be the subject of a future study.

Both Poiseuille-Couette flow and Poiseuille flow were found to result in potentially damaging stresses. The distribution of stresses on the particles showed that particles have had a stochastic exposure to stresses and these stresses could cause damage at different times during the movement of the particles. Higher stresses were observed for particles injected close to the channel wall, and exposure to lower stresses was observed for particles released at the center of the flow field. A model for vWF damage, similar to the power law models for hemolysis [47, 48], could be developed by determining both the appropriate exponent for the dependence on the time of exposure and the exponent for dependence on the observed stress. However, using a deterministic model to predict the stresses either based on wall shear stress or based on average stresses would not be an accurate approach. Instead, a stochastic model for the level of stresses and the probability of the vWF to be in regions of low or high shear stresses would be needed, with data similar to those presented herein. Further research to predict the shear stress distribution as a function of the flow conditions on the vWF particles is necessary.

As already mentioned in the Introduction, the mechanism of unraveling within the protein surrogate particle in our computations was not simulated–the details of the trajectories of such surrogate particles are calculated and the shear stresses along the individual trajectories of such surrogate particles are calculated and analyzed statistically. While changes at the molecular level may occur on the vWF molecules depending on the magnitude of the hydrodynamic stresses, these changes are assumed to not affect the fluid flow around them (i.e., they are assumed to be passive throughout the calculations). The reader can use our dimensionless data to obtain dimensional distributions of stresses and use other values of critical stress, based on any molecular model, to determine the percentage of vWF that may be compromised. Since the stress distribution results presented herein would apply to other cases of proteins with similar diffusivity as the vWF, or for small colloidal particles or micelles with high enough Schmidt numbers, one could also apply the present results to more general situations. The difference would be to determine from published work in the literature the level of the critical shear stress needed to modify the structure of such small particles or macromolecules.

## Supporting information

S1 File Data for average shear stresses in Poiseuille flow. (XLSX)

S2 File Data for average shear stresses in Poiseuille-Couette flow. (XLSX)

S3 File Data for the stress distribution in Poiseuille flow. (XLSX)

S4 File Data for the stress distribution in Poiseuille-Couette flow. (XLSX)
